# Uncovering miRNA–Disease Associations Through Graph Based Neural Network Representations

**DOI:** 10.3390/biomedicines14020289

**Published:** 2026-01-28

**Authors:** Alessandro Orro

**Affiliations:** Institute of Biomedical Technologies CNR, Via Fratelli Cervi 93, 20054 Segrate, Italy; alessandro.orro@cnr.it

**Keywords:** microRNA, miRNA–disease association, graph neural network

## Abstract

**Background:** MicroRNAs (miRNAs) are an important class of non-coding RNAs that regulate gene expression by binding to target mRNAs and influencing cellular processes such as differentiation, proliferation, and apoptosis. Dysregulation in miRNA expression has been reported to be implicated in many human diseases, including cancer, cardiovascular, and neurodegenerative disorders. Identifying disease-related miRNAs is therefore essential for understanding disease mechanisms and supporting biomarker discovery, but time and cost of experimental validation are the main limitations. **Methods:** We present a graph-based learning framework that models the complex relationships between miRNAs, diseases, and related biological entities within a heterogeneous network. The model employs a message-passing neural architecture to learn structured embeddings from multiple node and edge types, integrating biological priors from curated resources. This network representation enables the inference of novel miRNA–disease associations, even in sparsely annotated regions of the network. The approach was trained and validated on a dataset benchmark using ten replicated experiments to ensure robustness. **Results:** The method achieved an average AUC–ROC of ~98%, outperforming previously reported computational approaches on the same dataset. Moreover, predictions were consistent across validation folds and robustness analyses were conducted to evaluate stability and highlight the most important information. **Conclusions:** Integrating heterogeneous biological information and representing it through graph neural network representation learning offers a powerful and generalizable way to predict relevant associations, including miRNA–disease, and provide a robust computational framework to support biomedical discovery and translational research.

## 1. Introduction

MicroRNAs (miRNAs) are short, endogenous RNA molecules, usually 19–25 nucleotides long, that play a key role in regulating gene expression [1,2,3]. They act by incorporating into the RISC complex, which then binds to target messenger RNAs (mRNAs) at the 3′ untranslated regions (UTRs) through sequence complementarity, leading to gene silencing [4]. The resulting interaction normally leads to the inhibition of the target mRNA [5], although cases of translational activation have also been found in the literature [6]. In this way a single miRNA is able to regulate hundreds of gene transcripts, and, on a genome-wide scale, it is estimated that miRNAs are able to control expression of up to 60% of genes in the human genome [7], affecting virtually every physiological process. Starting from the initial discoveries of lin-4 and let-7 [8], the number of known miRNAs has increased rapidly in recent years, with the latest miRbase (Release 22.1) counting about 38,000 entries [9], underlining the evolutionary conservation and functional significance of these regulators. Dysregulation of miRNAs is associated with the pathogenesis of numerous complex human diseases [10], in particular in cancer [11], cardiovascular [12], neurodegenerative [13], and metabolic diseases [14]. For these reasons, identifying specific miRNA–disease associations (MDAs) is a useful step for understanding disease mechanisms and for developing novel therapeutic strategies [15]. Limitations of experimental approaches (PCR and high-throughput sequencing), which are typically resource-intensive, expensive, and time-consuming on a large scale [16], have motivated the development of computational methods to predict potential miRNA–disease associations (MDAs) [17,18].

Many approaches exploit the large amount of public data available today (like HMDD V2.0/V3.0 [19,20], dbDEMC [21], and miR2Disease [22]), relying on the widely accepted principle that functionally similar miRNAs are likely to be associated with diseases exhibiting similar phenotypes [23,24]. Early computational methods to predict MDAs can be broadly categorized into similarity-based approaches and modern machine learning/deep learning approaches. Early similarity-based approaches inferred MDAs by leveraging known interactions between miRNAs and their target genes, or between target genes and diseases. These methods often suffered from incomplete and noisy miRNA–target interaction datasets. This category includes models based on Random Walk over protein–protein interaction (PPI) networks [25] and methods like miRPD [26], which use intermediate networks to identify functional links between miRNAs and diseases.

To overcome these limitations, more sophisticated similarity-based network models were developed [27,28,29,30,31,32,33], integrating miRNA functional and disease semantic or phenotypic similarities with known MDAs. Approaches such as HDMP [34] relied on local similarity metrics, which proved ineffective for diseases without any known associated miRNAs (“new diseases”). This motivated the development of global network methods, for example, those employing the Random Walk with Restart (RWR) algorithm (RWRMDA [35], MIDP/MIDPE [36]). By traversing the entire network, RWR provides a global view of connectivity, significantly improving performance.

Further improvements integrated Gaussian Interaction Profile (GIP) Kernel similarity with functional and semantic similarity. Methods in this category, including WBSMDA [37] and HGIMDA [38], enabled the calculation of similarity for new entities (miRNAs or diseases) without prior associations, representing a significant advance toward predicting associations for both novel miRNAs and novel diseases.

Modern machine learning (ML) techniques provided more powerful tools to approach MDA prediction [39,40,41]. They range from supervised classifiers like Support Vector Machines (SVMs) [42] and Restricted Boltzmann Machines (RBMs) [43] to semi-supervised methods. A critical challenge for supervised learning is the difficulty in accurately obtaining reliable negative MDA samples. Addressing this, semi-supervised models like RLSMDA (Regularized Least Squares [44]) and Matrix Completion (MC) methods, such as MCMDA [45] were proposed. MCMDA, for instance, is highly efficient, operating only on the known positive MDA matrix by leveraging the assumption that the underlying adjacency matrix is low-rank, thereby inherently avoiding the need for negative samples. The high predictive power of MC methods was demonstrated by MCMDA, which achieved high AUC (87.49%) and a strong confirmation rate (up to 90% of top 50 predictions for diseases like prostate neoplasms). More recently, ensemble learning approaches such as ELMDA [46] have been proposed, which do not rely on known associations to calculate miRNA and disease similarities and use multi-classifier voting for prediction, achieving an average AUC of 92.29% on HMDD v2.0, confirming the potential of ensemble strategies in accurately predicting disease-associated miRNAs.

The continuous development of these models now involves various forms of Deep Learning and Network Embedding and Graph Attention Networks (GAT) [47,48,49] to capture complex, non-linear relationships within the integrated biological data.

Despite advances in computational prediction of miRNA–disease associations, key challenges remain. In particular, integrating heterogeneous biological data and capturing complex, non-linear relationships across miRNAs, diseases, and associated patterns is still difficult. Furthermore, limitations in data completeness and the dynamic nature of biological networks constrain model generalizability. Graph-based approaches, especially those leveraging message passing on heterogeneous networks, offer a natural framework to address these issues by propagating information across nodes and edges of multiple types, effectively learning embeddings that encode functional and phenotypic similarities.

In this work, we propose a Heterogeneous Graph Neural Network (GNN) that models miRNA–disease associations by leveraging a multi-node, multi-edge approach to integrate diverse sources of biological information. Similar to state-of-the-art GNN approaches for miRNA–disease prediction, including hypergraph convolution and attention-aware architectures, our model learns structured embeddings directly from the heterogeneous network, preserving relational information that is often lost in vectorized or engineered feature representations. Our framework differs from prior methods in its use of edge-type-specific message-passing layers and node-specific transformations, which enable effective propagation of functional signals across complex biological entities such as miRNAs, diseases, genes, and expression patterns. These mechanisms allow the network to capture non-linear dependencies, leading to robust prediction of miRNA–disease associations. Comparison with existing methods demonstrates improved predictive performance in terms of AUC-ROC.

## 2. Materials and Methods

### 2.1. Dataset

The dataset used in this study integrates multiple layers of biological information derived from curated repositories, ontology mapping, and sequence-level analyses. Experimentally validated miRNA–disease associations were obtained from the HMDD database [19,20] (version 2, 3.2, and 4). All miRNA identifiers were manually curated to ensure consistency across resources, including resolving deprecated or ambiguous names, normalizing letter case, and harmonizing naming conventions. The nucleotide sequences of all miRNAs were retrieved from miRBase [9].

Disease names reported in HMDD were manually normalized to match DisGeNET [50] terminology. This included removing formatting inconsistencies, resolving synonyms, and applying uniform rules before mapping each disease to its corresponding UMLS Concept Unique Identifier (CUI) [51]. Based on these CUIs, disease–gene associations were obtained from DisGeNET and represented as binary vectors indicating the presence or absence of gene relationships for each disease.

Similarity graph between miRNAs were computed using pairwise sequence alignments obtained with the well-known Needleman–Wunsch method [52]. Two miRNAs were considered similar if their alignment identity score was greater than 60%, generating a binary miRNA–miRNA adjacency matrix. Furthermore, k-mer frequency vectors (k = 2 and 3) were extracted to obtain sequence-based embeddings. Finally, short sequence motifs of length 4 were computed to derive the miRNA–pattern matrix, providing an additional sequence-derived relational layer.

**Figure 1:**
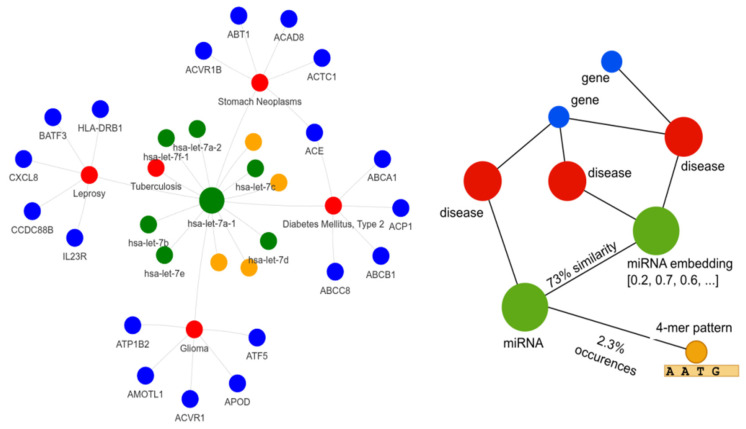
Graphical representation of selected portions of the heterogeneous biological network used in this study. Nodes represent miRNAs, diseases, genes, and sequence-derived patterns, while edges encode biologically meaningful relationships, including miRNA–disease associations, disease–gene links, miRNA–miRNA sequence similarity, and miRNA–pattern connections. Node colors indicate entity types. The right panel highlights a representative miRNA-centered subnetwork, where the central green node denotes a target miRNA, red nodes correspond to associated diseases, blue nodes to genes linked to those diseases, and orange nodes to sequence patterns connected to the miRNA, illustrating the multi-relational structure exploited by the model.

For HMDD version 4, the final dataset consists of 1183 miRNAs and 2114 distinct diseases, collectively forming 24074 miRNA–disease positive associations (0.96% matrix density). After harmonizing disease names with DisGeNET, we obtained a set of 6356 genes, which resulted in 18653 disease–gene associations mapped through UMLS CUIs. Sequence alignment produced miRNA–miRNA adjacency with 209186 entries, while the k-mer analysis yielded a structured representation of each miRNA through 80 embedding features. The extraction of motifs of length 4 generated 256 distinct patterns, leading to moderately dense adjacency matrices: 73,515 miRNA–pattern associations (~24%). Table 1 describes the details of graph size for the three versions of HMDD datasets. Figure 1 provides a graphical representation of a selected portion of the data graph for illustrative purposes, highlighting the structure and relationships among miRNAs, diseases, genes, and sequence-derived patterns.

**Figure 2:**
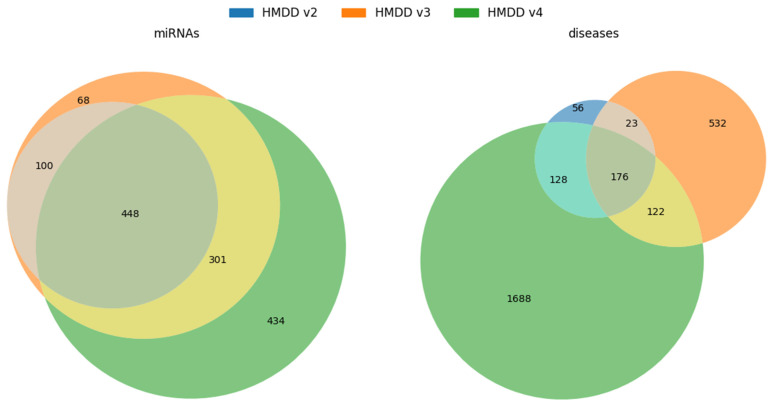
Venn diagrams showing the overlap of miRNAs (**left**) and diseases (**right**) across three versions of the HMDD database (v2, v3, and v4). The diagrams illustrate the number of shared and unique elements in each dataset version.

Analysis of the Venn diagrams (Figure 2) reveals that successive HMDD versions contain an increasing number of miRNAs and diseases. Notably, a substantial portion of the entries in earlier versions is retained in later releases, reflecting continuity and expansion of the curated data.

### 2.2. Graph Neural Network Architecture

**Figure 3:**
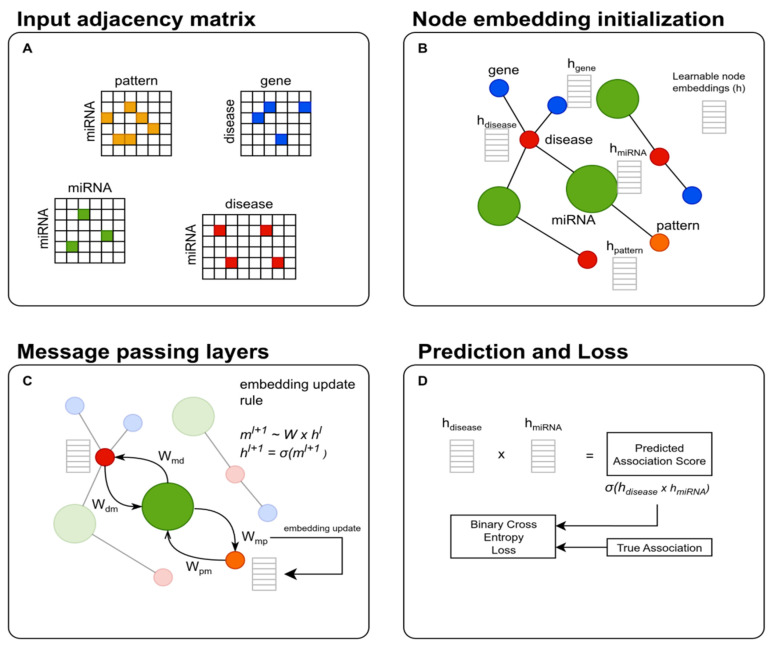
Overview of the heterogeneous graph neural network architecture for miRNA–disease association prediction. The model represents biological entities as nodes of four types (miRNAs, diseases, genes, and sequence patterns) connected by multiple biologically meaningful relations. (**A**) Each relation type is encoded as a sparse adjacency matrix, defining the heterogeneous graph structure. (**B**) The corresponding heterogeneous graph is constructed, where nodes are initialized with learnable embeddings. (**C**) Relation-specific message passing is performed using dedicated transformation matrices for each directed edge type, allowing information to propagate across heterogeneous neighbors. At each layer, node embeddings are updated by aggregating transformed messages from typed neighbors and applying a non-linear activation function. (**D**) After *L* message-passing layers, the final miRNA and disease embeddings are combined through a dot-product decoder to produce a prediction logit for each miRNA–disease pair. The predicted logits are optimized using a binary cross-entropy loss with logits, comparing the model predictions against ground-truth association labels.

We model the prediction of miRNA–disease associations using a heterogeneous graph neural network based on message passing [53]. The heterogeneous graph includes multiple node types, each associated with its own feature space, and multiple edge types capturing the biological relations among them (Figure 3). For each edge type e, the network learns a distinct linear transformation $W_{e}$, that governs how messages are propagated across that relation. Let G=(V,E) denote the resulting heterogeneous graph, where the each node v∈V has its own type (miRNA, disease, gene, and sequence pattern) and each edge e∈E has a type representing the nodes it connects (see Figure 3).

For a node v∈V its embedding at layer l is computed using a message-passing rule:

(1) $${E_{v}}^{l+1}={W_{0}}^{l}\cdot {h_{v}}^{l}+\frac{1}{\left|N(u,\;e)\right|}\cdot \sum_{e\in E}\;\sum_{u\in N(v,\;e)}\;{W_{e}}^{l}{h_{u}}^{l}$$

(2) $${h_{v}}^{l+1}=\sigma ({E_{v}}^{l+1})$$

where N(v,e) is the set of neighbors of v under relation e, and σ is a non-linear activation function, usually the *ReLU* function.

This mechanism allows the model to integrate heterogeneous biological signals-sequence-derived miRNA features, similarity relations, and gene-level mechanistic information—into a unified latent representation. This architecture enables end-to-end learning of latent biological relationships across the heterogeneous network.

After the message-passing layer L, the model computes node embeddings for miRNAs and diseases, and the association score for each miRNA–disease pair is then predicted by computing the dot product of the corresponding embeddings.

(3) $$S_{miRNA,disease}=\sigma (h_{miRNA}\cdot h_{disease})$$

During training, only edges of the miRNA–disease type contribute to the supervised loss defined by the binary cross-entropy calculated for the true (y) and predicted ($\hat{y}$) associations (edge) of a given batch B:

(4) $$loss=\frac{1}{\left|B\right|}\sum_{e\in B}\;y_{i}\cdot log(\sigma ({\hat{y}}_{i}))\;+\;(1-y_{i})\cdot log\left(\sigma \left({1-\hat{y}}_{i}\right)\right)$$

Nonetheless, all other edge types influence the node embeddings through relation-specific message passing, enabling the model to combine sequence-derived miRNA features, similarity networks, and gene-level signals into a unified latent representation.

Each node type in the heterogeneous graph is represented by a learnable embedding, which was initialized as a one-hot identity vector. These embeddings are updated during training through node-specific linear layers and multi-relational message passing, allowing the model to capture complex dependencies between miRNAs, diseases, and genes. No edge features are used; the relational structure is conveyed solely through the graph topology.

### 2.3. Training and Validation

The prediction of miRNA–disease associations is formulated as a binary link prediction problem on a heterogeneous graph. Known experimentally validated miRNA–disease associations are treated as positive labels, while negative examples are sampled from unannotated miRNA–disease pairs, as detailed below.

The evaluation follows a 10-fold cross-validation scheme, repeated 10 times with different random partitions to ensure robustness. In each fold, the set of miRNAs is randomly divided into a training and a validation subset. All nodes and all edges not involving miRNA–disease associations remain visible in both splits in order to preserve the global structure of the heterogeneous graph.

For the separation of miRNA–disease edges, all associations involving validation miRNAs are removed from the training graph, and symmetrically, all associations involving training miRNAs are removed from the validation graph. As a consequence, during validation the model is required to predict disease associations for miRNAs that were completely unseen during training, relying solely on message passing over the remaining graph structure and heterogeneous biological relations.

Positive samples correspond to all known miRNA–disease associations present in HMDD within the corresponding split. Negative samples are not defined as all unknown associations, but are instead randomly sampled from miRNA–disease pairs not reported in HMDD, following a standard negative sampling strategy for association prediction tasks. This avoids the unrealistic assumption that all unannotated pairs are true negatives and mitigates the strong class imbalance characteristic of miRNA–disease datasets. During training, negative samples involve only training miRNAs and exclude all known positive associations. During validation, negative samples involve only validation miRNAs and are explicitly constructed to exclude any miRNA–disease pair that is annotated as positive in HMDD. This ensures that no true positive associations are incorrectly treated as negatives and prevents label leakage between training and validation.

A similar masking strategy is applied to pattern–disease associations to avoid indirect information leakage through sequence-derived features. Specifically, pattern–disease edges are included in the training graph only if the pattern is connected to the disease through at least one miRNA–disease association belonging to the training set. If a pattern is linked to a disease exclusively through a validation miRNA–disease association, the corresponding edge is removed from the training graph (and symmetrically for the validation graph).

Each fold is trained independently using the Adam optimizer, with early stopping based on the validation loss. The training loss is computed only for miRNA–disease edges, while all other edge types contribute to learning through relation-specific message passing, enabling the model to integrate heterogeneous biological information while being evaluated under a strict and leakage-free generalization setting.

All diseases considered during validation have been previously observed in the training set; the model is thus evaluated on predicting novel miRNA–disease associations rather than on disease cold-start scenarios.

### 2.4. Evaluation Metrics

Performance was primarily assessed using AUC-ROC, which is the standard evaluation metric in MDA prediction due to the highly imbalanced nature of MDA datasets (<3% of positive samples). For comparison with other approaches, we additionally computed the area under the precision–recall curve (AUPR), Precision, Recall, and F1-score, defined as follows:

$$precision=\frac{TP}{TP+FP}$$

$$recall=\frac{TP}{TP+FN}$$

$$F1=2\cdot \frac{precision\cdot recall}{precision+recall}$$

## 3. Results

We first evaluated the proposed heterogeneous graph neural network on two benchmark datasets, namely HMDD v4.0, the most recent release, and HMDD v3.2 and v.2, widely used in prior computational studies, to facilitate direct comparison with the state of the art. All experiments were performed using the 10-fold cross-validation strategy described in Section 2.3, with the entire evaluation repeated 10 times using different random partitions.

Across all repetitions on HMDD v4.0, the presented model achieved an average AUC-ROC of ~98% and an AUPR of ~95%, demonstrating strong discriminative capability also in the presence of class imbalance. Similar results were obtained on HMDD v3.2, where the AUC-ROC reached ~97–98% and the AUPR remained consistently above 94%.

**Figure 4:**
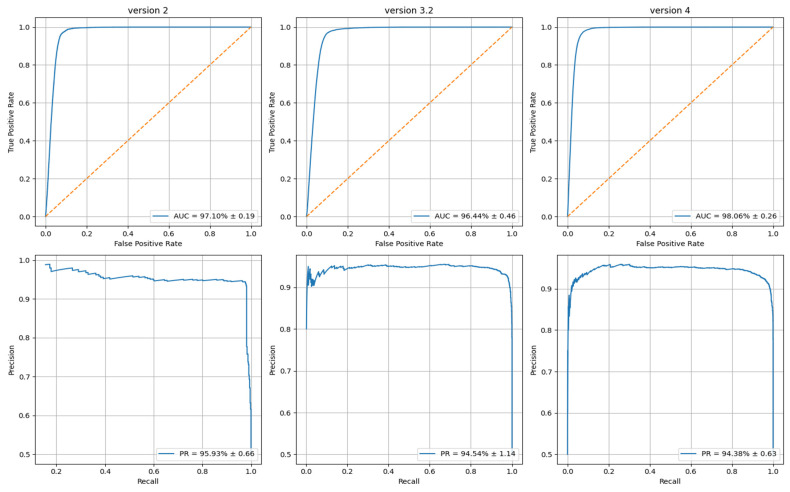
Receiver Operating Characteristic (ROC) and precision–recall (PR) curves obtained for the three evaluation datasets (HMDD v3.2, HMDD v4.0 and the combined heterogeneous dataset). Each curve represents the mean performance aggregated over all 10 × 10 replicated experiments, while the shaded regions denote the corresponding confidence bands. The tight variability observed across replications indicates the high stability of the model. The legend reports, for each dataset, the average AUC and AUPR (±their variance), confirming consistently strong predictive accuracy across all evaluation settings.

Figure 4 reports the mean ROC and PR curves aggregated over all replications. The narrow confidence bands observed in both curves indicate high stability across validation folds and independent experiments.

### 3.1. Comparison with Existing Methods

To position the presented approach to current computational models, we compared it against several representative methods evaluated on HMDD v2. We first considered four widely used and powerful machine learning methods that are known to be able to handle complex features, but still constrained to vectorized feature representations: Support Vector Machine (SVM), a margin-based classifier effective in high-dimensional settings; Gradient Boosting Decision Trees (GBDT), a sequential ensemble of decision trees using boosting to reduce errors; Random Forest (RF), an ensemble of decision trees; and eXtreme Gradient Boosting (XGBoost), a regularized boosting method offering strong predictive performance.

Traditional machine learning methods remain highly effective for structured biological prediction tasks, especially when relying on engineered similarity features or association profiles. Nevertheless, their inherent tabular representation of features hinders their ability to capture heterogeneous, multi-relational graph structures. This limits their capacity to exploit the full topology of miRNA–disease–gene–pattern networks—an aspect naturally handled by graph neural architectures.

Next, we included in the comparison six more specialized tools: MDA-CF [54], which leverages weighted hypergraph-based generalized matrix factorization to integrate multi-omics features of microbes and drugs, effectively predicting novel microbe-drug associations; TCRWMDA [55], employing hypergraph-based logistic matrix factorization to capture higher-order relationships between metabolites and diseases, enabling accurate identification of disease-related metabolites; WBSMDA [47], an attention-aware multi-view graph convolutional network combined with hypergraph learning to model miRNA–disease associations by integrating multiple similarity networks and fusing node information from diverse perspectives; ABMDA [56], which explores miRNA-mediated mechanisms underlying disease progression and drug resistance, providing experimentally informed predictions of functional miRNA–disease links; ICFMDA [57], a computational framework exploiting functional similarity and network inference to uncover potential miRNA–disease interactions; and ELMDA [46], an ensemble learning approach that does not rely on known associations to calculate miRNA and disease similarities, combining multiple classifiers via voting to robustly predict disease-related miRNAs across diverse validation settings.

Compared to the most competitive methods—MDA-CF (AUC 92.58%) and ELMDA (AUC 92.29)—our heterogeneous graph-based approach improves performance by a substantial margin, highlighting the benefits of: integrating heterogeneous biological relationships (miRNA–miRNA, disease–gene, miRNA–pattern, pattern–disease), Using message-passing to propagate functional signals across the network, and learning embedding representations directly from multiple node and edge types, rather than depending on pre-defined similarity kernels. These results indicate that the proposed model captures non-linear relationships more effectively than similarity-based or feature-engineering-based models.

Table 2 reports the performance metrics of the methods compared in this study, including AUC and AUPR and, when available, Precision, Recall, and F1-score.

### 3.2. Analysis of Newly Predicted Associations

**Figure 5:**
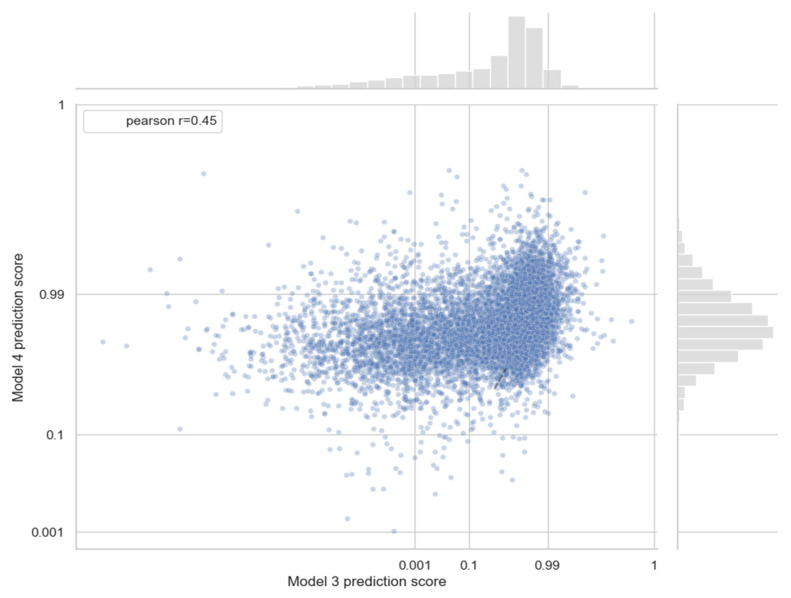
Comparison of prediction scores from Model 3 and Model 4 for selected miRNA–disease associations. Each point represents a miRNA–disease pair included in the analysis. The x-axis shows the prediction scores from Model 3, trained on HMDD v3.2, and the y-axis shows scores from Model 4, trained on the more complete HMDD v4.0 dataset. The Pearson correlation coefficient (r = 0.45) is reported in the legend, indicating moderate agreement between the two models while also revealing differences in predictions for novel associations.

In order to evaluate the ability of our models to predict novel miRNA–disease associations, we performed a comparative analysis using two graph neural models trained on two different versions of the HMDD dataset: Model 3 was trained on HMDD v3.2, containing only the associations known at that time, whereas Model 4 was trained on the more comprehensive HMDD v4.0, which includes additional associations reported after v3.2. Both models were then applied to predict association scores for all new miRNA–disease pairs, those not previously observed by Model 3. Since Model 3 is built on a smaller knowledge base, we expect it to perform less accurately. The Pearson correlation between the prediction scores of the two models shows a moderate correlation across all pairs (r ≈ 0.45), indicating that Model 3 is partially able to anticipate novel associations present in version 4 (Figure 5). Moreover, the AUCROC for Model 3 considering only the previously unseen positive associations was 89%, indicating that the model successfully discriminates against the majority of novel miRNA–disease links, further supporting its ability to anticipate associations absent from the training dataset.

**Figure 6:**
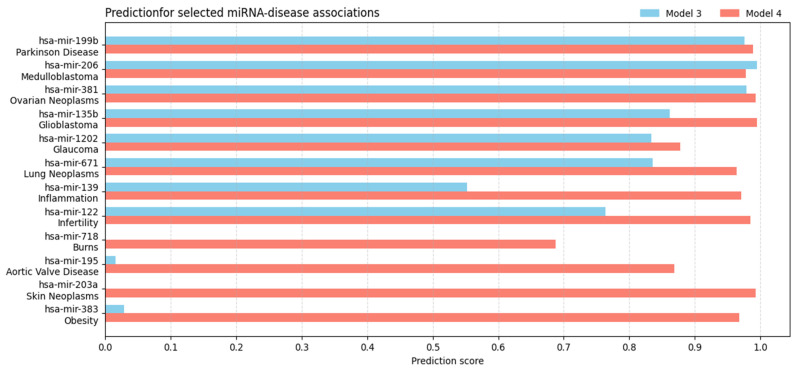
Bar plot comparing the prediction scores of Model 3 (trained on HMDD v3.2) and Model 4 (trained on HMDD v4.0) for a selected subset of positive miRNA–disease associations. Each row represents a single miRNA–disease pair, with the left bar indicating the score from Model 3 (blue) and the right bar the score from Model 4 (red). This visualization highlights both cases where Model 3 anticipates associations later reported in HMDD v4.0 and cases where the two models diverge, illustrating the models’ predictive behavior across heterogeneous scenarios.

To further analyze these differences, we examined a subset of representative positive associations and visualized the corresponding prediction scores from both models (Figure 6). Beyond simply contrasting the two score distributions, several patterns emerge: in many cases Model 4 assigns consistently higher confidence, reflecting the additional knowledge introduced in HMDD v4.0, while a number of pairs show near-identical scores, indicating that Model 3 successfully anticipates future annotations. Conversely, a few outliers (~15%) exhibit substantial divergence (absolute score difference greater than 0.5) between the two models, suggesting either overgeneralization by Model 3 or revised evidence incorporated in the updated dataset.

### 3.3. Ablation Analysis

Our heterogeneous graph neural network integrates multiple node types and relation-specific message passing to learn latent embeddings for miRNAs, diseases, genes, and sequence patterns. While the network is trained using the full set of edges, we observed that the model’s performance remains largely stable even when certain edge types are removed or perturbed. This indicates that the learned node embeddings capture significant information from node features themselves, and that the graph structure primarily provides additional contextual information rather than being strictly necessary for high predictive performance. Consequently, the predictive accuracy of miRNA–disease associations is robust with respect to partial or noisy graph information.

On the other hand we observe a different picture when the graph is perturbed before training. Specifically, ablating certain edge types prior to model training leads to significant drops in predictive performance, highlighting the importance of relational information during embedding learning. The quantitative effects of these pre-training ablations are reported in Table 3, showing that edge information is crucial for guiding the model to capture biologically meaningful associations.

In addition to edge-level ablations, we performed a disease-level holdout analysis to assess the model’s ability to generalize to unseen diseases. For each target disease, all miRNA–disease associations involving that disease were removed from the training set, while no negative samples were generated for the held-out disease, thereby preventing any form of data leakage. The model was then retrained on the reduced dataset and evaluated exclusively on the associations of the held-out disease.

This analysis was conducted on a representative subset of 35 diseases. Model performance was quantified using the area under the ROC curve (AUC), and the impact of disease removal was assessed by analyzing the difference between baseline and held-out performance ($\Delta AUC=AUC_{base}-AUC_{drop}$).

**Figure 7:**
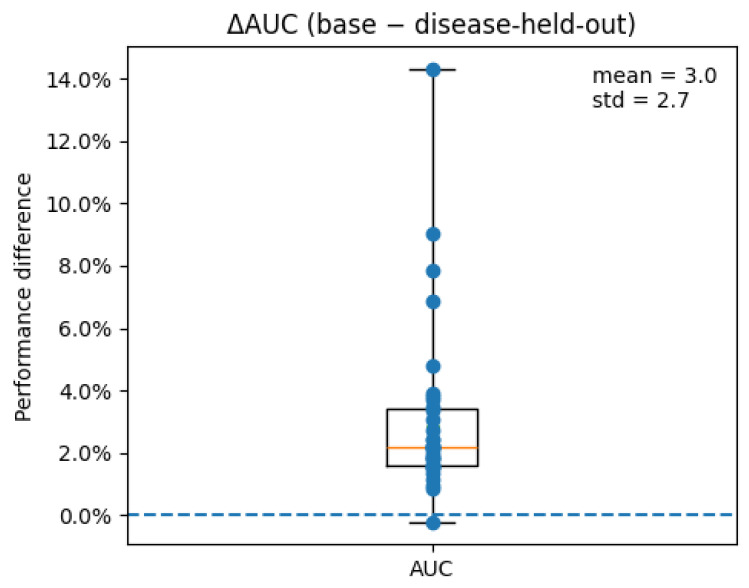
Distribution of the performance difference ΔAUC obtained by holding out entire diseases during training. Each data point corresponds to one disease removed from the training set.

As shown in Figure 7, holding out an entire disease leads to a consistent but moderate reduction in predictive performance. On average, the AUC decreases from 0.97 to 0.94 across the evaluated diseases, corresponding to a mean ΔAUC of approximately 0.03 (standard deviation ≈ 0.027). Despite this drop, performance remains well above random expectation, indicating that the learned embeddings retain substantial predictive power even when a disease is completely excluded from training. These results demonstrate that the proposed model is able to generalize to new disease contexts and that its predictions are not driven by data leakage from disease-specific associations.

### 3.4. Biological Interpretation of Selected miRNA–Disease Predictions

In order to provide an external and biologically meaningful assessment of the model predictions, we focused on a representative subset of high-confidence miRNA–disease associations among the top-ranked results. The selected examples (see Table 4) were prioritized based on the presence of independent evidence from published studies, which we verified to be absent from any other HMDD reference for the same miRNA–disease pair, allowing us to qualitatively evaluate the biological relevance of the predicted associations in terms of known pathways, target genes, and disease mechanisms.

For example, hsa-miR-99b was predicted to be associated with Wilms Tumor in our dataset. Independent evidence from a recent study showed that hsa-miR-99b-5p expression is significantly down-regulated in renal cancer tissues compared to adjacent normal kidney, and in silico analysis of its targets suggests involvement in angiogenesis-related pathways such as VEGF signaling. Although this study focused on renal carcinoma, the documented role of miR-99b-5p in tumor-related pathways supports the biological plausibility of the predicted association with Wilms Tumor. Moreover, in our predictions, hsa-let-7e was correctly associated with knee osteoarthritis. Beyond its established down-regulation in KOA patients [60], further independent evidence shows that hsa-let-7e-5p is part of a circulating miRNA signature linked to osteoarthritis phenotypes in a cohort of facet osteoarthritis patients, and that its predicted gene targets are enriched in a broad range of signaling pathways implicated in joint tissue pathology, based on interactome analysis [61]. Together, these findings support the biological plausibility of the predicted association between let-7e and knee osteoarthritis.

The miRNA hsa-miR-152-3p was predicted to be associated with spinal cord injuries in our dataset. Beyond its established association in HMDD [62], independent evidence indicates that hsa-miR-152-3p is upregulated in postmenopausal women with osteoporotic vertebral fractures [63]. Bioinformatic analysis suggests that hsa-miR-152-3p regulates key genes involved in bone matrix production and osteogenic differentiation, including WNT10B, ITGA5, ITGA9, COL2A1, and COL4A1, and modulates signaling pathways such as ECM-receptor interaction and stem cell pluripotency, highlighting a potential role in spinal tissue homeostasis and repair mechanisms.

The miRNA hsa-miR-208a was predicted to be associated with osteosarcoma in our dataset. Beyond its established association in HMDD [64], independent evidence demonstrates that hsa-miR-208a-3p is up-regulated in osteosarcoma tissues and promotes proliferation, migration, and invasion of osteosarcoma cells through targeting of PTEN, thereby implicating the PI3K/AKT signaling pathway in tumor progression [65]. These findings provide additional biological support for the plausibility of the predicted association, linking miR-208a to key pathways in osteosarcoma pathogenesis.

Finally, the extracellular vesicle-associated miRNA hsa-miR-210 was predicted to be associated with Parkinson Disease in our dataset. Independent evidence from studies of exosomal miRNAs in PD patients supports the involvement of EV-contained miRNAs in disease processes, including mechanisms related to intercellular transport of genetic material and modulation of neurodegenerative pathology such as α-synuclein aggregation, neuroinflammation, and neuronal stress responses, although specific targets for miR-210 in PD have not been comprehensively validated to date.

Analysis of a strong newly predicted association (false positive with respect to HMDD) shows that our model predicts a strong link between some miRNA that has been reported in the independent literature. For brevity we report only two cases. Independent experimental evidence supports association between hsa-miR-125b-1 and “Brain Ischemia”: hsa-miR-125b-5p has been shown [68] to protect neurons from ischemia–reperfusion injury by targeting ASIC1, a protein implicated in acidosis-induced neuronal death, thereby reducing neuronal damage in brain ischemia models. Moreover, pathway analysis of predicted gene targets suggests involvement in apoptosis regulation, inflammatory response, and neuroprotective signaling pathways, further supporting the biological plausibility of the predicted miRNA–disease association. This example illustrates the potential of the model to uncover biologically meaningful associations beyond existing database annotations. Finally, the association between hsa-mir-1193 and obesity has been reported in a study of bovine intramuscular fat deposition that shows that miR-1193 is upregulated in tissues with higher fat content and is part of a set of miRNAs identified as novel regulators of adipogenesis and lipid metabolism. In that study, differentially expressed miRNAs and their predicted target genes were associated with pathways involved in adipocyte differentiation and lipid homeostasis, as revealed by Gene Ontology and KEGG enrichment analyses, supporting the potential involvement of miR-1193 in obesity-related fat accumulation processes (e.g., adipocytokine and lipid metabolic pathways) [69].

For the analysis in this session, to support the biological interpretation of selected miRNA–disease predictions, relevant publications were retrieved using the PubMed API and processed using an AI-assisted text mining approach (Python library ScispaCy version 0.5 [70]) to extract information on miRNA target genes and associated pathways, followed by manual curation to ensure accurate interpretation.

## 4. Discussion and Conclusions

In this study, we presented a heterogeneous graph neural network framework for predicting miRNA–disease associations, leveraging the rich relational structure inherent in biological networks. Biological entities such as miRNAs, genes, diseases, and sequence motifs are naturally represented as nodes in a graph, with interactions forming edges of multiple types. Traditional machine learning approaches, which rely on tabular representations of features, often struggle to fully capture these complex, high-order relationships. In contrast, graph-based models excel at encoding both local and global structural patterns, allowing the integration of multiple types of biological information—ranging from sequence-derived features to gene–disease associations—within a unified latent space. This capability enables the model to infer indirect associations, identify hidden patterns, and generalize to previously unseen nodes with high accuracy.

Message-passing mechanisms within the network allow for effective propagation and aggregation of information, ensuring that the contribution of neighboring nodes is weighted according to their relevance, while attention-based layers enhance interpretability and robustness. Our experiments demonstrated that the model consistently achieves high predictive performance across multiple HMDD datasets, with narrow confidence intervals, confirming stability and reproducibility. Ablation and perturbation studies further highlighted the model’s sensitivity to network structure and its robustness to small levels of noise, underscoring the importance of accurately modeling heterogeneous interactions.

Despite these promising results, several limitations remain. While the model integrates diverse sources of biological information, the construction of the graph relies on pre-defined similarity measures and curated associations, which may overlook emerging or context-specific relationships. Future work could explore alternative strategies for graph construction, incorporating additional biological knowledge such as miRNA–target gene interactions, expression profiles across tissues or conditions, and temporal dynamics of disease progression. Moreover, integrating multi-omics data or environmental factors could enrich node features and edge relationships, improving prediction accuracy and providing deeper mechanistic insights. Advances in graph neural network architectures, including more sophisticated message-passing schemes or hierarchical graph representations, also offer avenues for performance enhancement and better interpretability.

Overall, this study confirms the strength of graph-based learning for miRNA–disease association prediction, demonstrating that modeling biological entities and their relationships as a heterogeneous network allows for accurate, robust, and generalizable inference. The proposed framework not only achieves state-of-the-art performance compared with existing methods, but also provides a flexible and extensible approach for future investigations, supporting the discovery of novel associations and facilitating hypothesis generation in translational research.

## Figures and Tables

**Table 1:** Graph size summary for the three HMDD dataset versions. For each version, the number of miRNAs, diseases, genes, and patterns and the total number of edges in each relationship type are reported. Edge density is expressed as the percentage of observed associations over all possible associations.

	Version 2	Version 3.2	Version 4
*nodes*			
miRNAs	548	917	1183
diseases	383	853	2114
genes	6356	6356	6356
patterns (4-mers)	256	256	256
*edges*			
miRNA–disease	6331 (3.02%)	15,161 (1.94%)	24,074 (0.96%)
miRNA–miRNA similarity	58,814 (19.58%)	133,958 (15.93%)	209,186 (14.95%)
disease–gene	11,977 (0.49%)	13,683 (0.25%)	18,617 (0.14%)
miRNA–pattern	36,602 (24.27%)	58,695 (25.0%)	73,515 (26.09%)

**Table 2:** Comparison of different machine learning approaches proposed in the literature reported from [46]. The proposed approach (P.A. in the table) has been reported for all three versions of the dataset. All other methods are evaluated based on version 2.

Method	Precision	Recall	F1-Score	AUCROC	AUPR
SVM	83.69 ± 0.85	83.71 ± 1.43	83.70 ± 0.75	90.91 ± 0.31	90.57 ± 0.36
GBDT	83.69 ± 1.07	84.90 ± 0.57	84.29 ± 0.54	91.72 ± 0.34	91.38 ± 0.39
RF	84.24 ± 1.08	83.54 ± 1.31	83.88 ± 0.91	91.41 ± 0.49	91.23 ± 0.47
XGBoost	84.71 ± 0.90	84.86 ± 0.99	84.78 ± 0.76	91.91 ± 0.39	91.65 ± 0.45
ELMDA	84.85 ± 1.39	85.36 ± 1.01	85.10 ± 0.94	92.29 ± 0.35	92.17 ± 0.31
MDA-CF	-	-	-	92.58	-
TCRWMDA	-	-	-	92.09	-
WBSMDA	-	-	-	81.85	-
ABMDA	-	-	-	90.45	-
ICFMDA	N.A.	N.A.	N.A.	90.23	N.A.
P.A. v2	92.02 ± 0.90	96.19 ± 0.90	94.06 ± 0.63	97.10 ± 0.19	95.93 ± 0.66
P.A. v3	91.49 ± 1.09	94.79 ± 1.02	93.11 ± 0.92	96.44 ± 0.46	94.54 ± 1.14
P.A. v4	94.94 ± 0.57	90.56 ± 1.79	92.70 ± 1.01	98.06 ± 0.26	94.38 ± 0.63

**Table 3:** Quantitative effects of AUC decrease caused by dropping different edge types.

Dropped Edge	Decrease AUC
miRNA–miRNA similarity	5.4%
disease–gene	11.2%
miRNA–pattern	3.4%

**Table 4:** Representative examples of top-ranked miRNA–disease associations predicted by the model and supported by independent literature evidence. For each association, the table reports the corresponding HMDD reference (disease and publication) or “not disease” in the case of newly predicted association. Additional evidence from external published studies, highlighting known pathways, target genes, or disease-related biological mechanisms are reported in the column “Literature Evidence”.

miRNA	HMDD	Literature Evidence
*hsa-mir-99b*	Wilms Tumor [58]	Down-regulation of hsa-miR-99b-5p in renal cell carcinoma tissues compared to normal kidney, with potential involvement in angiogenesis pathways through targets such as VEGF and TIMPs [59].
*hsa-let-7e*	Osteoarthritis, Knee [60]	Independent study identified hsa-let-7e-5p among circulating miRNAs associated with osteoarthritis phenotypes, and pathway analysis of its predicted gene targets revealed enrichment in multiple signaling pathways relevant to joint disease biology [61].
*hsa-mir-152*	Spinal Cord Injuries [62]	Independent study identified hsa-miR-152 among circulating miRNAs associated with vertebral bone lesions, suggesting potential involvement in spinal tissue homeostasis and repair mechanisms relevant to spinal cord injury pathology [63].
*hsa-mir-208a*	Osteosarcoma [64]	Independent study shows that hsa-miR-208a-3p is up-regulated in osteosarcoma tissues and promotes proliferation, migration, and invasion of osteosarcoma cells by targeting PTEN, implicating the PI3K/AKT signaling pathway in tumor progression [65].
*hsa-mir-210*	Parkinson Disease [66]	Expression and potential regulatory roles of hsa-miR-210 in Parkinson’s Disease have been observed in extracellular vesicle studies; while direct mechanistic pathways in PD remain to be fully delineated, exosomal miRNAs in PD patients’ biofluids are increasingly linked to disease-relevant processes including dysregulated intercellular signaling, neuronal stress responses, and α-synuclein propagation (Parkinson’s pathology) via EV-mediated communication [67].
*hsa-mir-125b-1*	Brain Ischemia (newly predicted)	Independent studies report that hsa-miR-125b-5p is involved in neuroprotection and neuronal survival during brain ischemia, supporting the biological plausibility of the predicted association [68].
*hsa-mir-1193*	Obesity (newly predicted)	Recent studies, however, suggest that it may play a role in fat deposition and metabolic regulation, supporting the potential biological relevance of this newly predicted association [69].

## Data Availability

The original contributions presented in this study are included in the article. Further inquiries can be directed to the corresponding author.
